# Processing and Representation of Ambiguous Words in Chinese Reading: Evidence from Eye Movements

**DOI:** 10.3389/fpsyg.2016.01713

**Published:** 2016-11-03

**Authors:** Wei Shen, Xingshan Li

**Affiliations:** ^1^Key Laboratory of Behavioral Science, Institute of Psychology, Chinese Academy of SciencesBeijing, China; ^2^University of Chinese Academy of SciencesBeijing, China

**Keywords:** homonymy, polysemy, Chinese, reading, eye movements

## Abstract

In the current study, we used eye tracking to investigate whether senses of polysemous words and meanings of homonymous words are represented and processed similarly or differently in Chinese reading. Readers read sentences containing target words which was either homonymous words or polysemous words. The contexts of text preceding the target words were manipulated to bias the participants toward reading the ambiguous words according to their dominant, subordinate, or neutral meanings. Similarly, disambiguating regions following the target words were also manipulated to favor either the dominant or subordinate meanings of ambiguous words. The results showed that there were similar eye movement patterns when Chinese participants read sentences containing homonymous and polysemous words. The study also found that participants took longer to read the target word and the disambiguating text following it when the prior context and disambiguating regions favored divergent meanings rather than the same meaning. These results suggested that homonymy and polysemy are represented similarly in the mental lexicon when a particular meaning (sense) is fully specified by disambiguating information. Furthermore, multiple meanings (senses) are represented as separate entries in the mental lexicon.

## Introduction

Lexical ambiguity is one of several types of ambiguities and is widespread in human languages. There are two types of lexical ambiguities: homonymy and polysemy (Lyons, 1977). A homonymy is a word that has two or more distinctly semantic unrelated meanings^1^. For example, the lexical item *bank* carries two completely unrelated meanings: *a financial institution* and *the slope of land beside a body of water*. In contrast, polysemy describes a word having two or more closely related senses. For instance, the lexical item *paper* has multiple related senses (i.e., *writing material, essay*, or *newspaper*). According to relative meaning frequency, ambiguous words can be either balanced or unbalanced. Balanced ambiguous words have multiple meanings of relatively equal frequency, while unbalanced ambiguous words have one high frequency meaning (i.e., the dominant meaning) and one low frequency meaning (i.e., the subordinate meaning; Rayner and Duffy, 1986). The current study aimed to investigate the processing and representation of these two lexical ambiguities in Chinese.

Two different but closely related research questions regarding lexical ambiguity are how they are represented and how they are processed. Words are thought to be represented as lexical entries in the mental lexicon and each lexical entry contains the lexical information concerning the word, such as semantic, syntax, phonology, orthography, and so on (Levelt, 1989). Word processing refers to the access of these information, is thus a more dynamic process through which words are retrieved, activated, and accessed, and can be influenced by many factors such as word representation and experimental tasks. In considering these two processes, an interesting question arises about whether polysemous words are represented and processed similarly or differently compared to homonymous words.

Previous studies conducted on alphabetical languages have shown that multiple meanings of a homonymous word are represented as separate lexical entries in the mental lexicon. Some researchers found that words with multiple meanings (homonymy) facilitated word processing in isolated word recognition studies. When using a lexical decision task, some studies found that response times (RTs) for homonymous words were shorter than for unambiguous control words (Rubenstein et al., 1970; Jastrzembski, 1981; Kellas et al., 1988). These results were used to argue that different meanings of homonymous words are represented separately in the mental lexicon, and the probability of choosing one meaning representation from multiple meaning representations was higher than that for unambiguous words. However, Rodd et al. (2002) found slightly different results when they investigated the processing of homonymy using a lexical decision task. They manipulated the number of meanings of a homonymous word and found that RTs were longer for homonymous words with more unrelated meanings than those with fewer meanings. They argued that a homonymous word may have multiple distributed meaning representations, and this processing disadvantage was attributed to the difficulty in mapping onto one semantic representation. The competition among multiple lexical entries during the process of reaching one meaning representation delayed the word recognition process. Although the underlying mechanism of the ambiguity effect still remains controversial (i.e., whether the relationship among multiple meanings is cooperative or competitive in a lexical decision task), research so far shows agreement that multiple meanings of homonymous words are represented as separate lexical entries in the mental lexicon.

In addition to evidence from isolated word recognition studies, eye-tracking studies have also provided evidence supporting separate representation for homonymy. In such studies, homonymous words were usually preceded by either a neutral or a dominant (subordinate) biasing sentence context. Readers spent longer fixation times on a balanced homonymy than an unbalanced one in a neutral context (Onifer and Swinney, 1981; Rayner and Duffy, 1986). These results suggested that the equivalent meaning frequency activated two meanings of a balanced homonymous word almost to the same level so that the competition for identification between the two meanings delayed meaning access. These results suggested that different meanings of homonymy are separately represented.

Many recent studies have investigated whether polysemous words are represented and processed differently compared with homonymous words. Some studies showed that they are represented and processed differently. Klepousniotou (2002) observed an ambiguity advantage effect for polysemy in a cross-modal sentence-priming lexical decision task. In this task, participants heard a sentence manipulated to create bias for one particular meaning of an ambiguous word. Then they were asked to perform a lexical decision task on visual letter strings presented at the offset of the spoken sentence. Faster RTs were observed for polysemous words than control words, matched in frequency and length, but no ambiguity disadvantage was found for homonymy. Based on these findings, the authors argued that multiple meanings of a homonymy are represented as separate lexical entries in the mental lexicon, while polysemy is represented only as an underspecified lexical entry (Rodd et al., 2002; Klepousniotou and Baum, 2007; Klepousniotou et al., 2008).

The differences between homonymy and polysemy were also observed in sentence reading tasks. Frazier and Rayner (1990) found that when a subordinate-biased disambiguating sentence followed the target word, readers spent longer times in the disambiguating region of the sentence when the target words were homonymous words rather than control words. However, reading times for polysemous words were comparable to control words. The authors argued that meanings of homonymous words were represented separately and the dominant meaning was the default meaning in their initial processing. Hence, for homonymous words, semantic commitment is immediate, and retrieval of another meaning is necessary when the previously accessed meaning is contextually inappropriate. They also argued that the lack of difference between polysemous words and control words was due to an underspecified core sense representation, in which only an underspecified core sense was accessed when polysemous words were initially encountered, and specified senses of polysemous words could only be created and extended online.

In contrast, others studies have showed that, like homonymy, polysemy is represented as separate lexical items. Recently, Foraker and Murphy (2012) provided evidence for this view in sentence reading using eye tracking technique. They embedded polysemous words in sentences and manipulated the context presented prior to the target word to make it biased toward the dominant sense, the subordinate sense, or neutral. The target sentence following the context was also biased toward the dominant or the subordinate sense of a polysemous word. A consistent condition was created when both context and target sentences were biased toward the same sense, and an inconsistent condition was created when context and target sentences were biased toward different senses. Processing was much more difficult in the inconsistent condition than in the consistent one. More importantly, a sense-dominant effect was found in the neutral condition, as evidenced by the faster processing of target sentences biased toward the dominant sense, as opposed to those biased toward subordinate sense. These results suggested that the initial processing of polysemous words depends on sense frequency, and the dominant sense is the default sense of the word to be accessed. Therefore, the senses of polysemous words are also represented as multiple specified senses rather than as an underspecified core sense of the words (see also Klein and Murphy, 2001, 2002, for a similar finding).

Clearly, the issue of whether polysemous words are represented and processed similarly or differently compared with homonymous words is far from clear. It is important to note that contradictory findings about the representation and processing of homonymy and polysemy have come from different experimental tasks, and different tasks may investigate different processing stages of homonymy and polysemy. For example, a lexical decision task and a reading task may reflect different aspects of semantic processing of a word (e.g., there is also a semantic integration involved in a reading task). According to the assumption of constraint-based model (Spivey-Knowlton and Sedivy, 1995; Jurafsky, 1996), during sentence reading, readers would make use of any available source of information (e.g., contextual information, syntactic information) for comprehension. The context-sensitive model also assumed that contextual information plays important roles in the process of ambiguity resolution (Paul et al., 1992). If the preceding contextual information is strongly constrained to one meaning, the activation level of the meaning is much stronger, thus can be accessed quickly; otherwise longer processing time is required under a weakly-constrained contextual environment. In short, the contextual strength may also modulate the degree of meaning activity. Therefore, the observed differences between homonymy and polysemy in previous studies may not necessarily reflect the differences in semantic representation but the difference reflected by contextual strength. Meanings of homonymy are less semantically related than those of polysemy. It is much easier to distinguish two distinct meanings than to distinguish two highly related senses. Therefore, it is possible that in sentence reading, richer contextual information and a deeper semantic processing is needed for total semantic commitment for polysemy, but not for homonymy. To overcome these shortcomings, we investigated the processing of these two types of words both in sentence reading.

It is worth noting that most studies on the representation and processing of polysemous words have investigated alphabetical languages and less is known about how these two types of words are represented and processed in Chinese. Like English, Chinese has a large number of ambiguous words. For example, the word 火星 (*huo3xing1*) is a homonymous word in Chinese and has two distinct meanings depending on context: “*Mars”* or “*fire sparks.”* The word 框架 (*kuang4jia4*) is an example of a polysemous word that carries two semantically related senses. It could mean “*composition frame”* or “*shell frame”* depending on the context.

Most studies conducted on the Chinese language have focused on homonymous words and have showed that homonymous words are represented separately in the mental lexicon as in alphabetical languages (Ren et al., 2007, 2008; Lin and Ahrens, 2010). In contrast to homonymous words, the representation and processing of polysemy in Chinese is less investigated and little is known about whether these two types of lexical ambiguities are similar or different in Chinese.

It is well known that alphabetical and non-alphabetical language systems have important cross-language differences. For example, a Chinese character contains more semantic information than an English letter (Hoosain, 1991). In addition, while English readers rely more on structural information for semantic interpretation, Chinese readers rely more on context information during lexical ambiguity resolution (Ahrens, 1998; Samovar et al., 2010). Thus, context may play a more important role in lexical ambiguity resolution in Chinese and, consequently, ambiguous words in Chinese are processed and represented differently than in English. In light of these similarities and differences between Chinese and English, investigating the processing and representation of ambiguous words in Chinese is important for fully understanding the representation and processing of words across all languages.

The major goal of the current study was to explore whether homonymous words and polysemous words were represented and processed similarly or differently in Chinese reading. Addressing this question is important because it can improve our understanding of homonymy and polysemy in general, and also reveal how they are represented in the mental lexicon. In particular, we were interested in examining whether the differences observed between two lexical ambiguities in prior studies was due to representational differences. If the two kinds of words have similar mental representation, the differences observed in previous studies cannot be accounted for by mental representation differences. Investigating both homonymy and polysemy in the same study could reveal some more general rules governing word representation and processing.

In the current study, we embedded unbalanced ambiguous words (homonymy and polysemy) in sentences and manipulated different parts of the sentences to provide different contexts for ambiguous words. The first part of the sentence was manipulated so that the target word was given either no clear biasing information (i.e., the neutral context condition) or bias toward either the dominant meaning (i.e., the dominant context condition) or the subordinate meaning (i.e., the subordinate context condition) of the target word. The critical target words were followed by a disambiguating region favoring either the dominant meaning (i.e., the dominant disambiguating condition) or the subordinate meaning (i.e., the subordinate disambiguating condition). A consistent condition was created when the prior context and disambiguating regions favored the same meaning of an ambiguous word, and an inconsistent condition was created when the disambiguating region supported a different interpretation than the prior context.

If polysemy is represented only as a core sense, and a specific sense is created online according to the underspecified core sense account, then no sense competition (i.e., longer reading times in the disambiguating region) should be observed in the inconsistent condition. On the other hand, if meanings (senses) showed a competition effect in the inconsistent condition, this would be strong evidence supporting a similar representation for both homonymy and polysemy (i.e., separate lexical representation). In addition, the contrasting ambiguity effects and processing differences observed in previous studies could not be explained on the basis of the representational difference between homonymy and polysemy.

## Methods

### Participants

Thirty participants (20 females and 10 males) were recruited from universities in Beijing near the Institute of Psychology, Chinese Academy of Sciences. Each was paid 45 Yuan (~9 U.S. dollars) to participate in this experiment. All were native Chinese speakers and had normal or corrected-to-normal vision. The participants' ages were from 18 to 28 years (*M* = 22.3 years). This study was approved by the Ethics Committee of the Institute of Psychology, Chinese Academy of Sciences. Written informed consent was obtained from all participants.

### Materials

Two hundred potentially ambiguous words with two meanings were first selected from *The Contemporary Chinese Dictionary* (Chinese Academy of Sciences, 2005) and the *Modern Chinese Polysemous Dictionary* (Yuan, 2001). Fifteen participants (none of whom participated in the later formal experiment) were recruited to judge the semantic relatedness of the two meanings on a 7-point scale (1 = not related at all; 7 = strongly related). The same pool of participants were also instructed to choose the first meaning that came to mind of two given alternative meanings when they saw the target words. Finally, 72 biased homonymous words and 72 biased polysemous words were selected. The score of semantic relatedness obtained for homonymy (*M* = 2.72, *SD* = 0.64; range from 1.27 to 3.73) was significantly lower than that obtained for polysemy [*M* = 4.19, *SD* = 0.48; range from 3.40 to 5.33; *t*_(71)_ = 13.99, *p* < 0.001]. Furthermore, the dominant meanings (*M* = 83%, *SD* = 14%) of homonymous words were more frequently selected as the first meaning than the selection of subordinate meanings (*M* = 16%, *SD* = 14%). Moreover, the selection of the dominant sense of polysemous words (*M* = 77%, *SD* = 14%) as the first sense was also much higher than the selection of subordinate sense (*M* = 22%, *SD* = 14%). In addition, word frequencies of homonymous words (*M* = 17.17 per million, *SD* = 34.80) and polysemous words (*M* = 12.23 per million, *SD* = 26.10) were comparable, and no significant difference was detected (*t* < 1).

Each of the ambiguous words was embedded in a sentence to assess the processing of ambiguous words in sentence reading. All experimental sentences were composed of two parts: a prior context and a disambiguating region. The prior context was manipulated to bias the target word toward either the dominant meaning (dominant context condition), subordinate meaning (subordinate context condition), or no biased information (neutral context condition). The disambiguating region followed the prior context and was consistent with either the dominant or subordinate meaning of the ambiguous word. The design was a 2 (word type: homonymy and polysemy) × 3 (context: dominant, subordinate, and neutral) × 2 (disambiguation: dominant and subordinate) within-participant design. This design created 3 consistent (when both prior context and disambiguating region were biased toward the same meaning or when the disambiguating region was biased toward the dominant meaning in the neutral context) and 3 inconsistent conditions (when prior context and disambiguating region were biased toward two divergent meanings, or when the disambiguating region was biased toward the subordinate meaning in the neutral context). Sample sentence materials are shown in Table 1.

**Table 1:** **Example experimental sentence in different conditions**.

**Word type**	**Prior context**	**Disambiguating region**	**Example sentences**
homonymy	Dominant	Dominant	当宇航员小王看到火星出现的那一瞬间，不由得对宇宙的壮美惊叹不已。
			(When the astronaut saw [the] **Mars/fire sparks**, he felt amazed at the beauty of the universe.)
	Dominant	Subordinate	当宇航员小王看到火星出现的那一瞬间，不由得对将临的危险心生恐惧。
			(When the astronaut saw [the] **Mars/fire sparks**, he was worried about the danger of fire.)
	Subordinate	Dominant	当消防员小王看到火星出现的那一瞬间，不由得对宇宙的壮美惊叹不已。
			(When the fireman saw [the] **Mars/fire sparks**, he felt amazed at the beauty of the universe.)
	Subordinate	Subordinate	当消防员小王看到火星出现的那一瞬间，不由得对将临的危险心生恐惧。
			(When the fireman saw [the] **Mars**/**fire sparks**, he was worried about the danger of fire.)
	Neutral	Dominant	当富商李成楠看到火星出现的那一瞬间，不由得对宇宙的壮美惊叹不已。
			(When the businessman Li Chengnan saw [the] **Mars/fire sparks**, he felt amazed at the beauty of the universe.)
	Neutral	Subordinate	当富商李成楠看到火星出现的那一瞬间，不由得对将临的危险心生恐惧。
			(When the businessman Li Chengnan saw [the] **Mars/fire sparks**, he was worried about the danger of fire.)
polysemy	Dominant	Dominant	那位小说家说这个框架已经非常完美了，总体逻辑结构非常严谨清晰。
			(The novelist said the **composition frame**/**shell frame** was perfect, the logic was very clear.)
	Dominant	Subordinate	那位小说家说这个框架已经非常完美了，主体工程应该很快就能完工。
			(The novelist said the **composition frame**/**shell frame** was perfect, the project should be complete soon.)
	Subordinate	Dominant	那位工程师说这个框架已经非常完美了，总体逻辑结构非常严谨清晰。
			(The engineer said the **composition frame**/**shell frame** was perfect, the logic was very clear.)
	Subordinate	Subordinate	那位工程师说这个框架已经非常完美了，主体工程应该很快就能完工。
			(The engineer said the **composition frame**/**shell frame** was perfect, the project should be complete soon.)
	Neutral	Dominant	那个郭月阳说这个框架已经非常完美了，总体逻辑结构非常严谨清晰。
			(Guo Yueyang said the **composition frame**/**shell frame** was perfect, the logic was very clear.)
	Neutral	Subordinate	那个郭月阳说这个框架已经非常完美了，主体工程应该很快就能完工。
			(Guo Yueyang said the **composition frame**/**shell frame** was perfect, the project should be complete soon.)

English translations of the example sentences were given in parentheses, and words in bold were two meanings (senses) of the target word.

### Normative data on the meaning bias of prior context

Prior context biased readers toward a dominant, subordinate meaning, or was neutral. Names of professions were employed in prior context to increase the activation level of intended meanings in different context conditions. For instance, 宇航员 (*astronaut*) and 消防员 *(fireman)* were used to provide the dominant and subordinate context conditions, respectively, for the target word 火星 (*mars*/*fire sparks*). In the neutral context condition, prior context always included personal names, and meaning access was based exclusively on meaning frequency. To confirm the bias of the prior context, another 15 participants (none of whom took part in later formal experiments) were presented with sentence fragments preceding the target words (all three conditions: the dominant, subordinate, and neutral context condition) and were instructed to guess the meaning of the target words based on the prior context by making a 2-alternative forced choice. These prior contexts were modified as necessary until 70% of the participants agreed on the intended meaning of the prior context.

### Normative data on the biasing of disambiguating region

Eighteen participants were recruited to rate the biasing of the disambiguating region, and they were given a target word plus the disambiguation region following it. They were instructed to guess the meaning of the target word based on the following disambiguating region by making a 2-alternative forced choice. Results showed that the mean percentage of choosing an intended biasing meaning of all sentences was 93%, indicating that disambiguating regions were biasing enough to activate the intended meaning.

### Normative data on the predictability of target word

Another 15 participants were presented with prior context until the target word, then asked to write down a word that fit into the prior context. If the target word was given, then the predictability was 1; otherwise, the predictability was 0. Results showed that only one item was predicted by 80% of the participants and almost 90% of the target words were completely non-predictable. The mean predictability of target words was 0.02.

### Normative data on the readability of all sentences

To ensure that the whole sentence could be understood by readers, we recruited another 18 participants to rate the readability of the sentences. They were asked to read the sentences and to respond “yes” if they could understand it and “no” if they could not. The results showed that 96% of all sentences could be understood by readers.

### Apparatus

Participants' eye movements were recorded using an eye-tracking system. A chinrest was used to minimize head movement during the experiment. Participants read sentences (which were presented horizontally from left to right on a single line) on a 21-inch CRT monitor (resolution: 1024 × 768 pixels; refresh rate: 150 Hz) connected to a Dell PC. They were seated 58 centimeters away from the computer; at this distance, one character subtended a visual angle of ~0.6°. Viewing was binocular, but only the right eye was monitored.

### Procedure

Before the experiment, participants were given a brief introduction to the eye tracker as well as instructions for the experiment. The eye tracker was calibrated and validated at the beginning of the experiment. Participants were required to look at a white dot randomly presented in a horizontal middle line of the screen. Calibration was conducted as necessary during the experiment. The calibration error was smaller than 0.5° of the visual angle. Each participant read 144 sentences (72 containing homonymous words and 72 containing polysemous words). In addition, 144 filler sentences with the same sentence structure were included randomly among the experimental sentences to prevent participants from becoming aware of lexical ambiguity. All sentences were presented randomly. In order to familiarize participants with the experimental procedure, 10 practice sentences were presented before the test session. Participants were instructed to read these sentences silently for comprehension, and comprehension questions were asked after about 30% of the sentences were read. All sentences were displayed on one line on the computer screen. Participants were instructed to fixate on a white dot presented in the middle of the screen for drift check at the beginning of each trial. Next, a white square (1° × 1°) was presented at the position of the first character of the sentence before the sentence was shown. Participants pressed a button on the bottom box to start the next trial. The whole experiment lasted about 90 min with a few short breaks.

## Results

Mean accuracy of the comprehension questions was 93%, indicating that participants could understand these sentences well. Trials with blinks in the target-word region, pre-target-word, or post-target-word regions and more than three blinks were discarded from the analysis, resulting in 12% of the trials being excluded. In addition, fixations shorter than 80 ms or longer than 1000 ms were also excluded from our analyses. Prior to statistical analysis, trials with a reading time beyond three standard deviations for each participant and each condition were excluded from the analysis. Mean fixation durations in milliseconds and standard errors for each condition are reported in Table 2.

**Table 2:** **Eye movement measures in difference conditions**.

**Measures**	**Conditions**		**Target word region**		**Disambiguating region**	
	**Context**	**Disambiguating**	**Homonymy**	**Polysemy**	**Homonymy**	**Polysemy**
First pass time	Dominant	Dominant	286 (8)	282 (8)	1198 (30)	1236 (35)
	Dominant	Subordinate	280 (9)	289 (13)	1103 (39)	1180 (42)
	Subordinate	Dominant	297 (8)	285 (8)	1116 (43)	1117 (30)
	Subordinate	Subordinate	290 (8)	287 (8)	1133 (34)	1141 (40)
	Neutral	Dominant	287 (8)	286 (8)	1134 (37)	1176 (32)
	Neutral	Subordinate	284 (8)	294 (11)	1070 (36)	1064 (37)
Total time	Dominant	Dominant	362 (16)	359 (23)	1782 (50)	1867 (70)
	Dominant	Subordinate	439 (24)	384 (24)	2041 (68)	2035 (54)
	Subordinate	Dominant	404 (14)	395 (18)	1891 (54)	1945 (56)
	Subordinate	Subordinate	415 (20)	386 (17)	1867 (52)	1921 (49)
	Neutral	Dominant	404 (20)	399 (19)	1815 (46)	1798 (55)
	Neutral	Subordinate	427 (19)	410 (20)	1947 (50)	1905 (56)
Regression-out probability	Dominant	Dominant	–	–	0.62 (0.05)	0.59 (0.05)
	Dominant	Subordinate	–	–	0.72 (0.05)	0.64 (0.04)
	Subordinate	Dominant	–	–	0.69 (0.05)	0.69 (0.05)
	Subordinate	Subordinate	–	–	0.69 (0.05)	0.64 (0.04)
	Neutral	Dominant	–	–	0.67 (0.05)	0.59 (0.05)
	Neutral	Subordinate	–	–	0.72 (0.05)	0.67 (0.04)

Standard errors are reported in parentheses.

**Figure 1:**
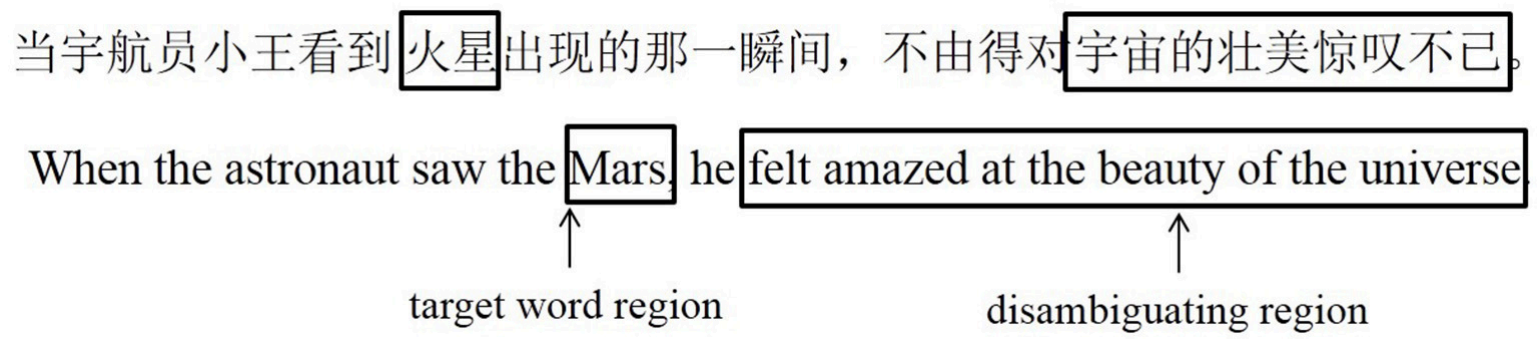
**An example of region of interest in a sentence**.

We mainly analyzed eye-movement measures in the target-word region (the two-character target word) and the disambiguating region (the disambiguating region). See Figure 1 for an example of how sentences were divided into regions.

We reported the following eye-movement measures: (1) first-pass time^2^ (the sum of all first-pass fixations on a word before moving to another word); (2) total time (the sum of all fixations on a word, including regressions); and (3) regression-out probability^3^ (regressions made from the current interest area to earlier interest areas). First-pass time reflects a relatively early lexical processing when readers encountered words during first-pass reading, while total time includes times for both earlier lexical processing and later semantic integration (Rayner et al., 2004). Regression-out probability also taps into a late processing stage which reflects an integration difficulty during reading and readers have to make regressions onto earlier parts to re-process. Eye movement measures were subjected to a series of repeated-measure analyses: 2 (word type: homonymy, polysemy) × 3 (prior context: dominant, subordinate, and neutral) × 2 (disambiguation: dominant disambiguating and subordinate disambiguating) ANOVA with context condition and disambiguating condition as within-participant factors and with participants (*F*_1_) and items (*F*_2_) as random factors.

A series of 2 (word type: homonymy, polysemy) × 3 (prior context: dominant, subordinate, and neutral) × 2 (disambiguation: dominant disambiguating and subordinate disambiguating) ANOVA were carried out with word type, context condition, and disambiguating condition as within-participant factors, and with participants (*F*_1_) and items (*F*_2_) as random factors.

### Target word region

For first-pass time, no main effects (*F*_*s*_ < 1) nor any interaction were found (*F*_s_ < 1). Also, we found that, in the dominant-subordinate condition, first-pass times on polysemous words were longer than that on homonymous words. However, this difference did not reach statistically significance [*F*_1_ < 1; *F*_2(1, 71)_ = 1.45, *p* = 0.23].

Total times on homonymous words (*M* = 409 ms, *SE* = 15 ms) were significantly longer than those on polysemous words [*M* = 389 ms, *SE* = 14 ms; *F*_1(1, 29)_ = 9.25, *p* = 0.005, $\eta_{\text{p}}^{2}$ = 0.24, MSE = 3723], and total times for the subordinate disambiguating condition (*M* = 410 ms, *SE* = 15 ms) were also longer than those for the dominant disambiguating condition [*M* = 400 ms, *SE* = 13 ms; *F*_1(1, 29)_ = 6.89, *p* = 0.01, $\eta_{\text{p}}^{2}$ = 0.19, MSE = 7067; *F*_2(1, 142)_ = 9.65, *p* = 0.002, $\eta_{\text{p}}^{2}$ = 0.06, MSE = 14,812]. Moreover, we found that total times on homonymous words were longer than those on polysemous words in the dominant-subordinate condition [*F*_1(1, 29)_ = 4.34, *p* = 0.05, $\eta_{\text{p}}^{2}$ = 0.13, MSE = 10,740; *F*_2(1, 71)_ = 4.77, *p* = 0.03, $\eta_{\text{p}}^{2}$ = 0.06, MSE = 23,382]. There was also a main effect of prior context [*F*_1(2, 58)_ = 3.89, *p* = 0.03, $\eta_{\text{p}}^{2}\;=$ 0.12, MSE = 4425; *F*_2(2, 284)_ = 2.63, *p* = 0.07, $\eta_{\text{p}}^{2}$ = 0.02, MSE = 15,442]. Total times for the dominant context (*M* = 386 ms, *SE* = 17 ms) were significantly shorter than those for the subordinate context [*M* = 406 ms, *SE* = 15 ms, *F*_1(1, 29)_ = 5.69, *p* = 0.02, $\eta_{\text{p}}^{2}$ = 0.16, MSE = 1998; *F*_2(1, 71)_ = 7.99, *p* = 0.006, $\eta_{\text{p}}^{2}$ = 0.10, MSE = 2819]. No difference was observed between the dominant (*M* = 386 ms, *SE* = 17 ms) and neutral context [*M* = 386 ms, *SE* = 17 ms, *F*_1(1, 29)_ = 2.29, *p* = 0.14; *F*_2(1, 71)_ = 1.01, *p* = 0.32], nor between the subordinate (*M* = 406 ms, *SE* = 15 ms) and neutral context (*M* = 386 ms, *SE* = 17 ms, *F*_s_ < 1).

Importantly, there was significant interaction between prior context and disambiguation [*F*_1(2, 58)_ = 2.77, *p* = 0.07, $\eta_{\text{p}}^{2}$ = 0.09, MSE = 7064; *F*_2(2, 284)_ = 3.09, *p* = 0.05, $\eta_{\text{p}}^{2}$ = 0.02, MSE = 150,385]. In the dominant context, total times on target words were longer when the disambiguating region favored the subordinate meaning (*M* = 412 ms, *SE* = 20 ms) than when it favored the dominant meaning [*M* = 361 ms, *SE* = 18 ms; *F*_1(1, 29)_ = 8.37, *p* = 0.007, $\eta_{\text{p}}^{2}$ = 0.22, MSE = 4688; *F*_2(1, 71)_ = 21.21, *p* < 0.001, $\eta_{\text{p}}^{2}$ = 0.23, MSE = 5007], indicating that processing of target words was hindered in the inconsistent condition. In the subordinate context, total times on target words when the disambiguating region favored the dominant meaning (*M* = 399 ms, *SE* = 14 ms) were indistinguishable from those that favored the subordinate meaning (*M* = 401 ms, *SE* = 16 ms; *F*_s_ < 1). In the neutral-context, although total times were numerically longer when the disambiguating region favored the subordinate meaning (*M* = 419 ms, *SE* = 16 ms) rather than the dominant meaning (*M* = 401 ms, *SE* = 17 ms), this difference was not statistically significant [*F*_1(1, 29)_ = 1.49, *p* = 0.23; *F*_2(1, 71)_ = 1.45, *p* = 0.23]. In addition, the three-factor interaction was also not significant, and no other main effect or interaction was significant.

### Disambiguating region

For first-pass time, main effects of prior context [*F*_1(2, 58)_ = 7.66, *p* = 0.001, $\eta_{\text{p}}^{2}$ = 0.21, MSE = 19,814; *F*_2(2, 284)_ = 3.88, *p* = 0.02, $\eta_{\text{p}}^{2}$ = 0.03, MSE = 106,421] and disambiguation [*F*_1(1, 29)_ = 4.39, *p* = 0.05, $\eta_{\text{p}}^{2}$ = 0.13, MSE = 46,931; *F*_2(1, 142)_ = 1.35, *p* = 0.25] were both significant. The interaction between prior context and disambiguation was also significant [*F*_1(2, 58)_ = 3.21, *p* = 0.05, $\eta_{\text{p}}^{2}$ = 0.10, MSE = 32,792; *F*_2(2, 284)_ = 5.02, *p* = 0.007, $\eta_{\text{p}}^{2}$ = 0.03, MSE = 94,109]. Simple-effect analysis showed that prior context affected first-pass time on the dominant disambiguating region [*F*_1(2, 58)_ = 6.25, *p* = 0.003, $\eta_{\text{p}}^{2}$ = 0.17, MSE = 12,255; *F*_2(2, 142)_ = 6.11, *p* = 0.003, $\eta_{\text{p}}^{2}\;=$ 0.08, MSE = 43,953]. First-pass times for the dominant disambiguating region were significantly longer in the dominant context (*M* = 1217 ms, *SE* = 25 ms) than in the subordinate context [*M* = 1117 ms, *SE* = 29 ms, *F*_1(1, 29)_ = 10.77, *p* = 0.003, $\eta_{\text{p}}^{2}$ = 0.27, MSE = 27,938; *F*_2(1, 71)_ = 11.02, *p* = 0.001, $\eta_{\text{p}}^{2}$ = 0.13, MSE = 64,839] or the neutral context [*M* = 1155 ms, *SE* = 26 ms, *F*_1(1, 29)_ = 8.05, *p* = 0.008, $\eta_{\text{p}}^{2}$ = 0.22, MSE = 14,161; *F*_2(1, 71)_ = 3.38, *p* = 0.07, $\eta_{\text{p}}^{2}$ = 0.05, MSE = 64,839]. In addition, first-pass times for the dominant disambiguating region were also longer in the neutral context (*M* = 1155 ms, *SE* = 26 ms) than those in the subordinate context (*M* = 1117 ms, *SE* = 29 ms), although the difference was only marginally significant in item analysis [*F*_1(1, 29)_ = 1.41, *p* = 0.24; *F*_2(1, 71)_ = 3.16, *p* = 0.08, $\eta_{\text{p}}^{2}$ = 0.04, MSE = 101,654]. Moreover, prior context also affected first-pass time in the subordinate disambiguating region [*F*_1(2, 58)_ = 3.71, *p* = 0.03, $\eta_{\text{p}}^{2}$ = 0.11, MSE = 14,048; *F*_2(2, 142)_ = 2.96, *p* = 0.05, $\eta_{\text{p}}^{2}$ = 0.04, MSE = 58,905]. First-pass times in the subordinate disambiguating region were significantly shorter for the neutral (*M* = 1067 ms, *SE* = 29 ms) rather than the dominant context [*M* = 1141 ms, *SE* = 34 ms, *F*_1(1, 29)_ = 5.09, *p* = 0.03, $\eta_{\text{p}}^{2}$ = 0.15, MSE = 32,437; *F*_2(1, 71)_ = 3.95, *p* = 0.05, $\eta_{\text{p}}^{2}$ = 0.05, MSE = 130,138] or for the subordinate context [*M* = 1137 ms, *SE* = 29 ms, *F*_1(1, 29)_ = 6.52, *p* = 0.02, $\eta_{\text{p}}^{2}$ = 0.18, MSE = 22,478; *F*_2(1, 71)_ = 4.15, *p* = 0.05, $\eta_{\text{p}}^{2}$ = 0.06, MSE = 127,999]. No difference was observed between the dominant and subordinate contexts (*F*_s_ < 1). These results suggest a wrap-up process during first-pass reading of the disambiguating region. When the integration was easier to process, readers would spend longer reading time in fully understanding this region. However, readers tended to make more regressions from this region when they encountered integration difficulty resulting in shorter reading times for the first pass time.

In addition, we found that in some conditions (e.g., dominant-subordinate condition) first-pass times in the disambiguating region were numerically longer for polysemous words than for homonymous words. However, this difference did not statistically significant [*F*_1(1, 29)_ = 3.00, *p* = 0.09, $\eta_{\text{p}}^{2}$ = 0.09, MSE = 29,672; *F*_2(1, 71)_ = 3.01, *p* = 0.08, $\eta_{\text{p}}^{2}$ = 0.04, MSE = 118,232].

For total time, there was a main effect of prior context [*F*_1(2, 58)_ = 4.15, *p* = 0.02, $\eta_{\text{p}}^{2}$ = 0.13, MSE = 31,085; *F*_2(2, 284)_ = 3.54, *p* = 0.03, $\eta_{\text{p}}^{2}$ = 0.02, MSE = 98,362] and a main effect of disambiguation [*F*_1(1, 29)_ = 13.12, *p* = 0.001, $\eta_{\text{p}}^{2}$ = 0.31, MSE = 72,933; *F*_2(1, 142)_ = 13.25, *p* < 0.001, $\eta_{\text{p}}^{2}$ = 0.09, MSE = 206,852]. Total times were longer when the disambiguating region favored the subordinate meaning (*M* = 1953 ms, *SE* = 42 ms) than when it favored the dominant meaning (*M* = 1850 ms, *SE* = 46 ms).

**Figure 2:**
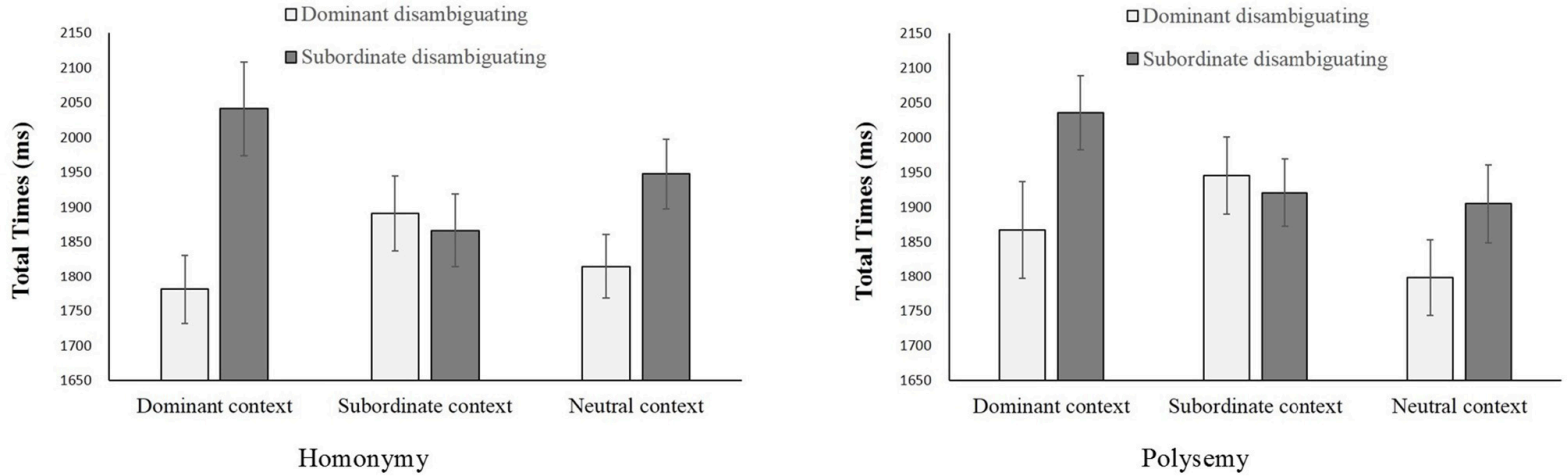
**Total times in the disambiguating region under different conditions by word type (homonymy, polysemy), prior context (dominant, subordinate, and neutral) and disambiguation (dominant disambiguating and subordinate disambiguating)**.

Although no three-factor interaction was found in the disambiguating region (*F*_s_ < 1), there was significant interaction between prior context and disambiguation [*F*_1(2, 58)_ = 8.20, *p* = 0.001, $\eta_{\text{p}}^{2}$ = 0.22, MSE = 52,953; *F*_2(2, 284)_ = 12.39, *p* < 0.001, $\eta_{\text{p}}^{2}\;=$ 0.08, MSE = 103,709] (See Figure 2 for total times under six conditions for homonymy and polysemy, respectively). Simple-effect analysis showed that prior context affected total times on the dominant disambiguating region [*F*_1(2, 58)_ = 5.79, *p* = 0.005, $\eta_{\text{p}}^{2}$ = 0.17, MSE = 18,828; *F*_2(2, 142)_ = 5.09, *p* = 0.007, $\eta_{\text{p}}^{2}$ = 0.07, MSE = 48,166]. Total times for this region were significantly longer in the subordinate context (*M* = 1918 ms, *SE* = 50 ms) than the dominant context [*M* = 1824 ms, *SE* = 54 ms, *F*_1(1, 29)_ = 12.74, *p* = 0.001, $\eta_{\text{p}}^{2}$ = 0.31, MSE = 20,957; *F*_2(1, 71)_ = 6.40, *p* = 0.01, $\eta_{\text{p}}^{2}$ = 0.08, MSE = 98,953] or the neutral context [*M* = 1806 ms, *SE* = 47 ms, *F*_1(1, 29)_ = 9.05, *p* = 0.005, $\eta_{\text{p}}^{2}$ = 0.24, MSE = 41,715; *F*_2(1, 71)_ = 9.56, *p* = 0.003, $\eta_{\text{p}}^{2}$ = 0.12, MSE = 86,406]. This result also suggested that meaning difficulty was encountered in the inconsistent condition. No difference was observed between the dominant and neutral contexts (*F*_s_ < 1).

Moreover, prior context also affected total times on the subordinate disambiguating region [*F*_1(2, 58)_ = 7.45, *p* = 0.001, $\eta_{\text{p}}^{2}$ = 0.20, MSE = 23,191; *F*_2(2, 142)_ = 10.49, *p* < 0.001, $\eta_{\text{p}}^{2}$ = 0.13, MSE = 54,444]. Total times on the subordinate disambiguating region were significantly longer for the dominant context (*M* = 2038 ms, *SE* = 56 ms) than the subordinate context [*M* = 1894 ms, *SE* = 40 ms, *F*_1(1, 29)_ = 11.17, *p* = 0.002, $\eta_{\text{p}}^{2}\;=$ 0.28, MSE = 56,146; *F*_2(1, 71)_ = 19.47, *p* < 0.001, $\eta_{\text{p}}^{2}$ = 0.22, MSE = 109,838] or the neutral context [*M* = 1926 ms, *SE* = 46 ms, *F*_1(1, 29)_ = 8.86, *p* = 0.006, $\eta_{\text{p}}^{2}$ = 0.23, MSE = 42,639; *F*_2(1, 71)_ = 10.06, *p* = 0.002, $\eta_{\text{p}}^{2}$ = 0.12, MSE = 112,334]. No difference was observed between the subordinate context and the neutral context [*F*_1(1, 29)_ < 1, *F*_2(1, 71)_ = 1.52, *p* = 0.221].

In addition, neither the interaction between word type and prior context [*F*_1(2, 58)_ = 1.03, *p* = 0.37; *F*_2(2, 284)_ = 1.27, *p* = 0.28], nor that between word type and disambiguation were significant (*F*_s_ < 1).

Regression-out probability was higher in the homonymy condition (*M* = 0.69, *SE* = 0.04) than the polysemy condition [*M* = 0.64, *SE* = 0.04; *F*_1(1, 29)_ = 8.02, *p* = 0.01, $\eta_{\text{p}}^{2}$ = 0.22, MSE = 291]. Furthermore, participants also made more regression-outs from the subordinate disambiguating region (*M* = 0.68, *SE* = 0.04) than the dominant disambiguating region [*M* = 0.64, *SE* = 0.04; *F*_1(1, 29)_ = 12.39, *p* = 0.001, $\eta_{\text{p}}^{2}$ = 0.29, MSE = 103; *F*_2(1, 142)_ = 5.17, *p* = 0.02, $\eta_{\text{p}}^{2}$ = 0.04, MSE = 598].

Moreover, the interaction between prior context and disambiguation was also significant [*F*_1(2, 58)_ = 5.16, *p* = 0.01, $\eta_{\text{p}}^{2}$ = 0.15, MSE = 168; *F*_2(2, 284)_ = 3.94, *p* = 0.02, $\eta_{\text{p}}^{2}$ = 0.03, MSE = 609]. Simple-effect analysis showed that prior context significantly affected regression-out probability on the dominant disambiguating region [*F*_1(2, 58)_ = 6.85, *p* = 0.002, $\eta_{\text{p}}^{2}$ = 0.19, MSE = 90; *F*_2(2, 142)_ = 7.10, *p* = 0.001, $\eta_{\text{p}}^{2}$ = 0.09, MSE = 262]. Regression-out probability for the dominant disambiguating region was significantly higher in the subordinate context (*M* = 0.69, *SE* = 0.04) than the dominant context [*M* = 0.60, *SE* = 0.04, *F*_1(1, 29)_ = 17.66, *p* < 0.001, $\eta_{\text{p}}^{2}$ = 0.38, MSE = 131; *F*_2(1, 71)_ = 14.63, *p* < 0.001, $\eta_{\text{p}}^{2}$ = 0.17, MSE = 497] or the neutral context [*M* = 0.63, *SE* = 0.05, *F*_1(1, 29)_ = 5.44, *p* = 0.03, $\eta_{\text{p}}^{2}$ = 0.16, MSE = 225; *F*_2(1, 71)_ = 5.11, *p* = 0.03, $\eta_{\text{p}}^{2}$ = 0.07, MSE = 564]. No difference was observed between the dominant and neutral contexts (*F*_s_ < 1). In addition, no interaction was found among word type, prior context, and disambiguation (*F*_s_ < 1).

## Discussion

The current study investigated whether the processing and representation of homonymous words and polysemous words are similar or different in Chinese reading. We manipulated two different parts in a single sentence: (i) the prior context preceding target words, and (ii) the disambiguating region following the target words. We compared these two lexical ambiguity words (i.e., homonymy and polysemy) within one experiment.

We found similar data patterns for homonymy and polysemy. When the prior context and disambiguating region created bias toward the same meaning or sense (the consistent condition), readers spent less total time on the target word as well as on the disambiguating region and made fewer regressions out from the disambiguating region. However, when the prior context and the disambiguating region created bias toward divergent meanings (the inconsistent condition), longer total times on the target word and disambiguating regions, and more regression-outs were observed in the disambiguating region, as indicated by significant interaction between context and disambiguation. The observed significant interaction between prior context and disambiguation showed that different meanings (senses) tend to compete with each and the processing was hindered in the inconsistent condition.

These results showed that different meanings of a homonymous word are separately represented. In the inconsistent condition, readers had to retrieve another meaning when a contextually inappropriate meaning was previously accessed, indicating that different meanings were incompatible with each other. This finding was in line with results in studies of alphabetical languages, supporting a separate lexical account for homonymy (Rubenstein et al., 1970; Jastrzembski, 1981; Kellas et al., 1988). There was a similar data pattern from polysemy to homonymy. Total times were also shorter in the consistent condition rather than the inconsistent condition, indicating that different senses of polysemous words tended to compete with each other and thus delayed processing. Taken together, our data demonstrated that senses of polysemous words have salient and separate lexical representations in the mental lexicon, when different senses are fully disambiguated and specified by abundant context. This finding cannot be fully explained by the underspecified core sense representation account. According to this account, no individual lexical representation for the sense of polysemous words is represented in the mental lexicon. Senses of polysemous words are represented only as an underspecified core sense, and specific interpretations of polysemous words rely on sense extension online through a series of lexical rules. Therefore, no sense selection is necessary and no sense competition would occur in the processing of polysemy even if senses were fully specified. However, results from the current study showed that this was not the case. In our study, significant interaction was again found in the disambiguating region, and sentence processing was more efficient in the consistent condition and less efficient in the inconsistent condition. Furthermore, word type did not interact with the other two factors to influence the processing. Thus, not enough evidence was found to argue for different lexical representations for homonymy and polysemy.

In addition, we also found longer first-pass times in the disambiguating region in the consistent condition than that in the inconsistent condition for both homonymy and polysemy. The increased processing time observed under the consistent condition suggested a wrap-up process (Just and Carpenter, 1980) and an integration process is needed before the end of the sentence. However, in the inconsistent condition, readers were aware of integration difficulties and the integration processing was blocked, thus they had to go back to earlier parts of sentences to reprocess, resulting in shorter reading times in the disambiguating region. This wrap-up effect was observed on first-pass time indicating that different meanings (senses) were already initiated by two sources of contextual information (i.e., prior context and disambiguating region) at a relatively early processing stage.

It should be noted that some eye movement measures were different between homonymy and polysemy in some conditions. For example, for polysemous words, in the dominant-subordinate condition, first-pass times on target words were longer than that for homonymous words and a similar pattern was also observed in the disambiguating region. However, these differences did not reach statistical significance in both regions. In addition, we also found that total times on homonymous words were significantly longer than those on polysemous words in dominant-subordinate condition. These differences cannot be easily interpreted as that the processing of the processing of homomymous words was different from the processing of polysemous words. Especially given that the two types of ambiguous words were embedded into two different sentence frames in our current study, thus those differences may be caused by sentence frames. Again, we did not find a three-factor interaction involving word type, thus these “differences” cannot necessarily reflect the processing difference between homonymy and polysemy.

Although semantic relatedness among senses of polysemy is higher than relatedness among the senses of homonymy, these senses are fully specified when a sentence provides abundant context information. For instance, a Chinese polysemous word 创伤 can refer to psychological trauma as well as physical wound. These interpretations refer to two totally different ideas in spite of their semantic relatedness. Therefore, it was difficult for readers to integrate two different senses during sentence reading.

From this aspect, the separate representation account and the underspecified core sense representation account may not be completely incompatible. The critical difference lies in whether the disambiguating information is abundant enough to specify one particular meaning (sense) of ambiguous words. It is highly likely that semantic competition occurs when different meanings (senses) are fully specified and activated by different disambiguating regions in one sentence (as in the current study). However, homonymy and polysemy may be processed differently in a situation in which meanings (senses) are underspecified or only partially specified when the contextual information is not abundant or biased enough. This under-specification of meanings (senses) could explain why different data patterns for homonymy and polysemy were found in previous studies (Frazier and Rayner, 1990; Rodd et al., 2002; Klepousniotou and Baum, 2007; Klepousniotou et al., 2008, 2012).

Frazier and Rayner's study found a garden-path effect on homonymy but not on polysemy when the disambiguating context followed target words (Frazier and Rayner, 1990). Based on this result, they proposed an underspecified core sense account for polysemy. According to the context-sensitive model as mentioned above, contextual strength can influence the meaning (sense) activation level of an ambiguous word (Paul et al., 1992). Therefore, one possible explanation for the observed lack of processing difference may be that their experimental sentences were not constrained or rich enough to activate one specific sense of polysemy. For example, it was not necessary to activate one specific sense of *newspaper* (a polysemous word) when reading a sentence like “Unfortunately the newspaper was destroyed, lying in the rain.” The two senses of the word “newspaper” were closely semantically related (“a physical paper” or “a business news publisher”), and the context preceding the word *newspaper* was not biasing enough to distinguish between the two senses. Different data patterns observed in their study may only reflect some processing differences rather than representational differences between the two. Therefore, the findings of Frazier and Rayner's study cannot provide firm evidence against the separate representation account of polysemy. On the other hand, our current data provide evidence that the two types of words were processed similarly when fully specified by disambiguating information. The processing was hindered when different meanings were initiated by the prior context part and the disambiguating region. Retrieving another meaning was necessary when an inappropriate meaning had been previously accessed.

Moreover, whether a meaning (sense) is fully specified can be modulated by the experimental tasks as well. Previous isolated word-recognition studies found different ambiguity effects for homonymy and polysemy and attributed this difference to inner representational difference between homonymy and polysemy. Difference in tasks employed in these studies might account at least partially for the differences in results. Our primary aim in this study was to explore whether the mental representations of homonymy and polysemy are similar or different when they are completely specified. The significant and stable interaction effect between context and disambiguation for total time in the disambiguation region in the current study provided firm evidence that the processing was facilitated in the consistent condition because readers could successfully integrate the initial selected meaning with the rest of the sentence, while processing was hindered in the inconsistent condition since different meanings were biased by prior context and disambiguating regions. These results provided some evidence that homonymy and polysemy had very similar lexical representations when they were fully specified by abundant context, with each meaning (sense) represented as a separate lexical entry in the mental lexicon. It should be noted that only biased polysemous words were included in our study, it still not clear whether biased and unbiased polysemous words have the same inner lexical representations in the mental lexicon (i.e., separate lexical representation). More studies are needed to further investigate this issue.

In the current study, we examined the representation of ambiguous Chinese words—specifically, those exhibiting homonymy and polysemy. We found a separate lexical representation for both homonymy and polysemy with their meanings (senses) were saliently represented separately in the mental lexicon, and this finding is in line with those of Klein and Murphy (2001, 2002). Although some similarities have previously been reported in the processing and representation of ambiguous words across languages, it is notable that these similarities concerning ambiguity appeared to have been established at the lexical level. Compared to English, ambiguity is known to occur even at the morphemic level in Chinese (Tsang and Chen, 2013). It is well known that most Chinese words are composed of two individual characters, and each character can correspond to more than one morpheme. For example, a Chinese character 打 has at least 20 meanings in the dictionary, However, this level of ambiguity can be resolved at once when the character forms a word with another character, such as 打鼓 (beat the drum), 打水 (fetch water). The core mechanism underlying resolution of sub-lexical ambiguity in Chinese still needs to be established in future studies.

In summary, we did not find evidence supporting a different lexical representation account for Chinese homonymous and polysemous words. Instead, our data suggest that they may have separate representations in the mental lexicon when they are fully specified by disambiguating information during sentence reading.

## Author contributions

Conceived and designed the experiments: WS, XL. Performed the experiments: WS. Analyzed the data: WS, XL. Wrote the paper: WS, XL.

### Conflict of interest statement

The authors declare that the research was conducted in the absence of any commercial or financial relationships that could be construed as a potential conflict of interest.

## Footnotes

^1^The term “meanings” here referred to multiple distinct and unrelated lexical entries of homonymy, while the term “senses” referred to multiple semantic related lexical entries of polysemy.

^2^Noted that this measure is generally termed as “gaze duration” when the region of interest is an individual word. For consistency, we used the term “first-pass time” here.

^3^This measure calculates regressions to the target-word region and other parts of the sentences.
